# Evidence for ADAR-induced hypermutation of the Drosophila sigma virus (Rhabdoviridae)

**DOI:** 10.1186/1471-2156-10-75

**Published:** 2009-11-26

**Authors:** Jennifer A Carpenter, Liam P Keegan, Lena Wilfert, Mary A O'Connell, Francis M Jiggins

**Affiliations:** 1 Institute of Evolutionary Biology, University of Edinburgh, West Mains Rd, Edinburgh EH9 3JT, UK; 2 MRC Human Genetics Unit, Western General Hospital, Edinburgh EH4 2XU, UK; 3 Department of Genetics, University of Cambridge, Downing Street, Cambridge CB2 3EH, UK

## Abstract

**Background:**

ADARs are RNA editing enzymes that target double stranded RNA and convert adenosine to inosine, which is read by translation machinery as if it were guanosine. Aside from their role in generating protein diversity in the central nervous system, ADARs have been implicated in the hypermutation of some RNA viruses, although why this hypermutation occurs is not well understood.

**Results:**

Here we describe the hypermutation of adenosines to guanosines in the genome of the sigma virus--a negative sense RNA virus that infects *Drosophila melanogaster*. The clustering of these mutations and the context in which they occur indicates that they have been caused by ADARs. However, ADAR-editing of viral RNA is either rare or edited viral RNA are rapidly degraded, as we only detected evidence for editing in two of the 104 viral isolates we studied.

**Conclusion:**

This is the first evidence for ADARs targeting viruses outside of mammals, and it raises the possibility that ADARs could play a role in the antiviral defences of insects.

## Background

Adenosine deaminases that act on RNA (ADARs) are RNA-editing enzymes that target regions of double stranded RNA (dsRNA), converting adenosine (A) to inosine (I) [1]. Because I base-pairs with cytidine (C), during reverse transcription these Cs are then base-paired with guanosine (G) when the second strand is made, and so editing events show up as changes from an A to a G when sequenced. Similarly, the conversion of A to I is read by the translation machinery as if it were guanosine, and so editing events alter the protein sequence encoded by edited mRNA (see [2] for review).

Previous studies have shown that ADARs act on dsRNA of ~100 bp or longer, modifying anywhere from a single base to up to 50% of the adenosines [3] and have a strong 5' neighbour preference (*A *= *U *>*C *>*G*), rarely targeting adenosines less than three nucleotides from the 5' terminus, or eight nucleotides from the 3' terminus [4,5].

In mammals [6], *Drosophila *[7] and squid [8] most of the ADAR-edited transcripts are expressed in the central nervous system and are thought to allow more than one protein to be produced from a single gene. Among these edited transcripts are genes involved in ion channels, including the glutamate-gated ion channel receptors (Glu-R) [6] and serotonin (5-HT2c) receptor in humans [9], and the glutamate-gated chloride-channels [10], calcium-channels [7] and sodium-channels [11] in *Drosophila*. ADAR-editing in these ion-channel transcripts generates huge protein diversity within the nervous system, for example, cacophony--a voltage-gated calcium channel in *Drosophila*--is edited at 10 different sites, potentially generating more than 1000 different isoforms [11].

Aside from editing mRNAs in the central nervous system, ADARs also target transposable elements [12,13] and RNA viruses: A to G mutations thought to be caused by ADARs have been found in vesicular stomatitis virus (Rhabdoviridae) [14], in three retroviruses; human immunodeficiency virus type 1 (HIV-1), rous-associated virus (RAV-1) and avian leucosis virus (ALV) [15-17]; two paramyxoviruses; human respiratory syncyial virus (RSV) and measles virus [18,19]; the polyoma virus (PV: Polyomaviridae) [20] and the hepatitis delta virus (HDV) [21]. In the case of HDV, the mutations occur at specific sites in the viral RNAs, but in most other viruses the A to G mutations can occur throughout the viral genomes, and typically involve the hypermutation of ~50% of As to Gs within a defined region. Although it is unclear whether this hypermutation is harmful to viruses, there is evidence that hyper-editing of viral transcripts prevents them from being transported into the cytoplasm [20]. Further to this, Scadden [22] describes a nuclease that cleaves edited dsRNA in *Drosophila*, providing the first tentative evidence for ADARs being involved in tagging viral RNAs for degradation.

In this study, we describe evidence for ADAR caused hyper-editing in the sigma virus--a negative sense RNA virus that is a pathogen of *D. melanogaster*. We found hypermutation of A to G in two viral lines, with the mutations being biased towards sites that are predicted to be preferred by ADAR. This is the first evidence to suggest that ADAR targets viruses in insects.

## Results and Discussion

### Results

#### Evidence of hypermutation in viruses

**Figure 1:**
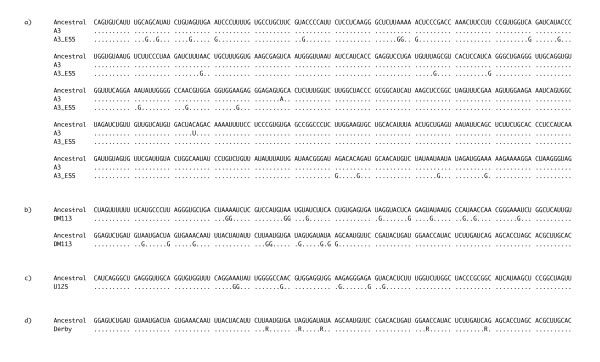
**Sequences of the gene encoding the PP3 protein of sigma virus that contain clusters of A to G mutations**. The sequences are shown in positive sense (the mRNA sequence rather than the genome sequence). (a) Isolates A3 and A3 E55 (2'916 - 2'316). These were laboratory maintained lines that were split from each other ~15 years ago. (b) The field isolate DM113 (3'194 - 2'954). (c) The field isolate U125 (2'693 - 2'574). (d) The field isolate Derby (3'074 - 2'954). This isolate was polymorphic for five A to G changes in the PP3 protein (these ambiguous bases are indicated by the wobble code R). In all the panels, sites that have not been mutated are represented by a period and the ancestral sequences were reconstructed by parsimony using a phylogeny of the viral sequences. The nucleotide locations refer to the position in negative sense genome of Genbank accession AM689308 (isolate A3).

We initially sequenced ~5,700 bp of the viral genome from two sigma virus lines (A3 and A3-E55), which shared a common ancestor about 15 years ago and found 25 nucleotide differences between them. Using an outgroup, we assigned these mutations to either the lineage leading to A3 or A3-E55 and found a significant difference in the number of mutations on the two lineages (χ_2 _= 11.58, *d.f. *= 1, *P *< 0.001), with 21 of the 25 mutations occurring on the A3-E55 lineage. All 21 of these changes were from A to G in the positive sense replication intermediate, and all but one were clustered within a 565 bp region of the gene encoding the PP3 protein (Figure 1; permutation test of clustering: P < 0.0001). Thirteen of these 21 changes were nonsynonymous (i.e. they change the amino acid sequence encoded by the gene). The four mutations on the other A3 lineage were not clustered, nor were they changes from A to G (A3 mutations: two G to A, one C to U and one C to A).

Because ADARs are known to edit As to Gs in other RNA viruses, we examined the mutations we found in our viral line to see if they were characteristic of ADAR-type editing. ADARs prefer to edit adenosines that don't have a 5' guanosine [4,5]. To test whether preferred sites were more likely to have mutated in our viral sequence, we reconstructed the sequence of the common ancestor of our two strains using an outgroup and compared the frequency of changes at preferred sites with those at un-preferred sites. We found that within the 571 bp hypermutated region, none of the 32 As that were preceded by a G had been mutated. However, 20 of the 109 As at preferred sites had been mutated to a G. This paucity of guanosines preceding mutated sites is significant when compared to unchanged sites (Fisher's Exact Test *P *= 0.007, see Table 1), suggesting ADARs are indeed responsible for the mutations.

**Table 1:** P-values from Fisher's exact test calculated for the four viral isolates showing patterns of A to G hypermutations.

	Mutated As		Unmutated As		
Viral isolate	preferred site*	unpreferred site**	preferred site	unpreferred site	*p*-value
A3 E55	20	0	89	32	0.007
DM113	20	0	16	11	0.001
U125	4	1	1	5	0.08
Derby	5	0	8	7	0.114

*preferred sites for ADAR-induced hypermutations are As that are preceded by either an A, U or C.

**unpreferred sites for ADAR-induced hypermutations are As that are preceded by a G.

***unmutated sites are defined as As within the cluster of mutations that have not been mutated.

We went on to look for evidence of similar hypermutation events in 102 wild sigma isolates by sequencing ~5,100 bp region of the genome. We were interested in recent mutations that could have been caused by ADAR, so we examined only mutations that were found in just one of the viral sequences (singletons) and that were either A to G or T to C changes. In all 102 isolates, we found only two isolates that had groups of A to G or T to C mutations that were significantly clustered: DM113 and Derby (permutation test of clustering: P < 0.0001 in both cases).

In the DM113 isolate, which has the greatest number of singletons of all 102 isolates, all 20 mutations (11 of the 20 mutations were non-synonymous) were from A to G in the positive sense replication intermediate, and all were clustered within a 146 bp region of the gene encoding the PP3 protein (Figure 1). Other than these changes, DM113 is identical in sequence to other closely related isolates. As before, we tested whether sites preferred by ADAR were more likely to have mutated, by reconstructing the ancestral sequence using an outgroup, and compared the frequency of changes at preferred sites with those at un-preferred sites. We found that there was a highly significant excess of mutations occurring at preferred sites (Fisher's Exact Test *P *= 0.001, see Table 1).

In the Derby isolate, all 5 mutations (4 of the 5 mutations were non-synonymous, with one introducing a stop-codon) were from A to G in the positive sense replicate intermediate, and all were clustered within a 65 bp region of the gene encoding the PP3 protein (Figure 1). However, this is a special case, as these five changes are polymorphic at these sites (both As and Gs are present in the RNA extracted from a single infected fly line), suggesting that only a proportion of the viral genomes have been edited. Again, we tested whether sites preferred by ADAR were more likely to have mutated, but found they were not (Table 1). Other than these two examples, there was a third case of suspected hypermutation. In the U125 isolate, which had the third highest number of singletons in our dataset, five of the 13 mutations were from A to G, and these five mutations were clustered within a 24 bp region of the gene encoding the PP3 protein (Figure 1). However, overall the 13 mutations were not significantly clustered (permutation test: P = 0.28), nor were the five A to G changes significantly more likely to occur at preferred sites (Table 1).

This approach of direct sequencing viral genes will only detect mutated viral RNA when the majority of viral copies in the fly have been mutated. However, it is unlikely, especially if these mutation are deleterious to the virus, that all viral copies will be simultaneously mutated. We therefore looked for mutated viral RNA present at a lower frequency. To do this we amplified the PP3 gene by PCR and cloned the PCR product from a subset of our wild viral isolates. However, we found no further evidence of hypermutation: across 86 clones (~600 bp in length and derived from 10 different viral strains) we found 14 mutations, all of which occurred only once. Of these, 6 of the 14 changes were in the direction of ADAR-editing (A to G and U to C), and 3 of these 6 changes were at sites preferred by ADAR (i.e. A not preceded by G, and Ts not followed by C).

#### Editing rates in sigma infected flies

**Figure 2:**
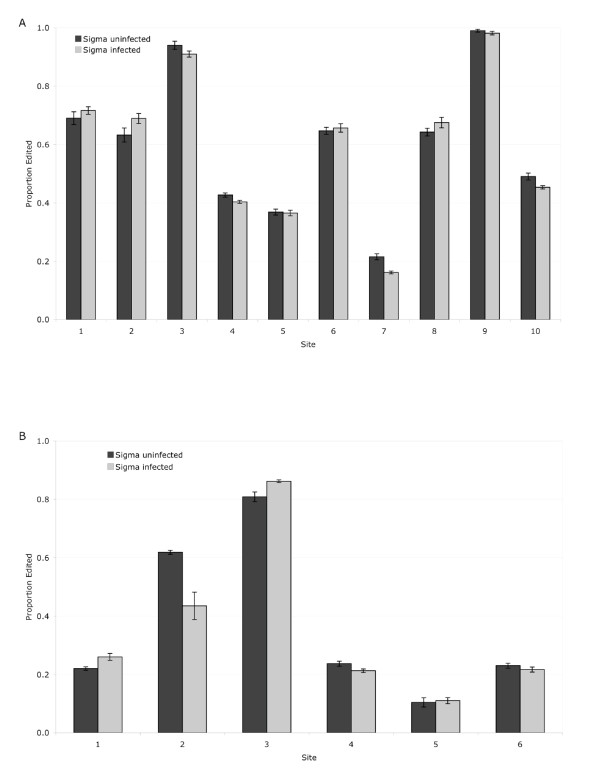
**Proportion of edited transcripts in two genes (Graph A: Nicotinic Acetylcholine Receptor a 34E (nAChR); Graph B: Resistant to dieldrin (Rdl) in Drosophila either infected with the sigma virus or uninfected**. nAChR is edited at 10 sites, Rdl is edited at 6. Graphs show means with standard errors.

In the final experiment, we examine whether the sigma virus is capable of suppressing ADAR-activity as a defence against the potentially harmful hypermutations. The rationale for this experiment is based on the observation that the sigma virus has the unusual property of paralyzing or killing flies when exposed to high concentrations of carbon dioxide. Intriguingly, flies that lack a functioning copy of the ADAR gene display a similar symptom, where they are paralysed following anoxia. If ADAR editing has an antiviral function i.e. introducing mutations that are deleterious to the virus, then we might expect the sigma virus to suppress ADAR activity. The suppression of ADAR activity might explain why sigma-infected flies become paralysed when exposed to carbon dioxide. To test this, we examined whether the rate at which fly mRNAs are edited by ADAR is altered by sigma virus infection. ADAR edits the *nAChR *transcript at 10 sites and the *Rdl *transcript at 6 sites [23]. However, there was no difference in the editing rates of either of these two genes in sigma-infected and -uninfected flies (Figure 2; *nAChR*: *F*_1,116 _= 0.386, *P *= 0.535; *Rdl*: *F*_1,55 _= 0.001, *P *= 0.969).

## Discussion

We have provided the first evidence from outside mammals to suggest that viruses can be hypermutated by host ADARs. The clustering of A to G changes observed in the laboratory viral lines are typical of mutations that occur as a result of RNA editing by ADARs, in that they are changes from A to G and occur at sites that don't have a 5' G [4,5]. Definitive evidence that ADAR is causing this hypermutation will require experimental verification, for example by showing that hypermutation does not occur in flies lacking ADAR.

The frequency of edited viral RNA is low, and we only found evidence for editing in two of 104 viral isolates. While this may reflect a low rate of editing, it is also possible that the edited RNA is rapidly degraded or hypermutations are deleterious and do not persist within the viral population. This is supported by the observation that the hypermutation events we observed caused numerous changes to the encoded protein sequence. If this is the case, RNA editing may be common but difficult to observe. This may explain why both of the editing events that we observed are in the PP3 protein that has no known function, while, no editing-events were found in the other genes--where hypermutations would likely be removed due to their harmful effects. Alternatively, this region of the genome may be edited more often because it is prone to form dsRNA. We may also lack statistical power to detect some editing events, as two other sequences showed patterns consistent with editing but were not significant.

Evidence of RNA-editing by mammalian ADARs has been found in a number of different viruses, including the vesicular stomatitis virus (VSV; Rhabdoviridae), which is a close relative of the sigma virus [14]--both are negative-sense RNA virus. An intriguing question is how ADARs are able to edit the genome of a negative-sense RNA virus? Our data suggests that in both of the hypermutation events that we found, it is the replication intermediate that was edited rather than the negative sense viral genome (the A to G changes all occur on the positive strand). This suggests that dsRNA is either formed between the viral genome and the complimentary replication intermediate, or is the result of secondary structure in the replication intermediate. However, it was recently shown that RNA viruses with negative sense genomes produce little or no dsRNA in vivo [24], so it is unclear how ADAR is able to edit VSV and the sigma virus. Editing not only requires the formation of dsRNA, but also requires ADAR and the virus to be present in the same cellular compartment. It is not known whether *Drosophila *ADAR is active in the cytoplasm, but our results suggest that it probably is, or the sigma virus enters the nucleus. One additional factor that may expose the sigma virus to editing by ADAR is that, like other Rhabdoviruses, it is known to infect the nervous system, where ADARs are known to be active [25].

Intriguingly, ADAR mutants have very similar phenotypes to sigma-infected flies, experiencing nervous-tremors similar to the tremors and paralysis experienced by sigma-infected flies after exposure to CO_2_. However, we found no evidence that viral infection alters the rate at which host mRNAs are edited, so it is unclear whether these two phenotypes are linked.

So why are viruses edited? In hepatitis delta virus, RNA editing is an essential process in the life-cycle of the virus; HDV hijacks ADARs, which edit a single site in the virus, switching production of a protein involved in virus replication to a protein involved in virion assembly [26]. However, this does not explain the function of the ADAR-induced hyper-editing found in other viruses, which show evidence of RNA-editing, but do not require editing to switch from one stage of their life-cycle to another. For these viruses, one possibility is that they get caught up with ADARs inadvertently, and the editing of viral genomes has no biological role.

Alternatively, editing events caused by ADARs might be part of an antiviral defence. Under this scenario, one possibility is that mutations introduced into viral genomes by ADARs could impact the efficiency of the viral multiplication process by altering either the stability of dsRNA formed during viral replication, or the sequence of viral mRNAs, so they no longer encode functional viral proteins [27]. There is also evidence in the polyoma virus that editing prevents viruses moving freely between host cells [20].

The second possibility is that the inosines introduced into viral RNA may act as a tag, marking the virus for subsequent degradation [22,28], although there is evidence that this is not always the case [29]. In support of this idea, an RNAse called Tudor-SN--a component of the RNAi silencing complex (RISC)--specifically degrades ADAR-edited RNA in *Drosophila *[22,28,30]. This raises the possibility of a link between RNAi, an important antiviral defence in flies, and ADAR editing. As evidence for this, a previous study has shown that many small-interfering (si)RNAs produced by the RNAi pathway have evidence of ADAR-editing [12]. However, another study has shown that edited RNA may sometimes be stable and retained in the nucleus [31], and so whether viruses are hyperedited by ADARs and fed into the RNAi pathway remains a contentious issue [32]. But evidence that ADAR can induce hypermutations in the sigma virus, as reported in this paper, demonstrates the importance of investigating the involvement of ADARs in anti-viral defences in both insect and mammalian models.

## Conclusion

In this study, we provide the first evidence that suggests ADARs target viruses outside of mammals, and it raises the possibility that ADARs could play a role in the antiviral defences of insects.

## Methods

We sequenced 5744 bp of the genome from two sigma virus lines (A3 and A3E55), supplied by Didier Contamine (Genbank accession numbers: A3; AM689308, A3E55; AM691026). These viral lines are a single wild collected isolate which was split between two separate fly lines and maintained at 20 degree Celsius for between 10 and 20 years. We then sequenced 5108 bp of the genome from 102 wild viral isolates collected from 8 populations in Europe and 2 populations in North America (L. Wilfert, unpublished). For details of sequencing methods see [33]. We examined sequences with greater than 5 singletons by eye for evidence of ADAR editing. Three sequences with suggestive evidence of hypermutation were found (Genbank accession numbers: DM113; GQ451694, U125; GQ451695 and Derby; GQ451693. We went on to test this statistically using a permutation test to investigate whether A to G, or T to C, mutations are clustered together in a sequence. First, we calculated the mean distance (number of nucleotides) between all the mutations in the sequence. We then permuted the order of the nucleotides and recalculated this statistic 10,000 times to generate a null distribution. The proportion of the permuted statistics that are less than the observed statistic was used as a *P*-value.

To detect mutated viral RNA present at low frequency in the sample, we amplified a region of the PP3 gene using the high fidelity polymerase (AccuPrime Pfx DNA Polymerase, Invitrogen, Paisley, UK), cloned the PCR product using a TOPO TA cloning kit (Invitrogen, Paisley, UK) and sequenced individual clones. Next, we tested whether viral infection alters the rate at which *Drosophila *mRNAs are edited. We extracted RNA from 10 day old flies that were either infected with the sigma virus (viral isolate AP30 [33]) or uninfected (but were otherwise genetically identical) [33]. cDNA was synthesised with MMLV Reverse Transcriptase (Promega, Madison, WI). Editing rates for *nAChR *and *Rdl *were measured examining the chromatograms following Sanger sequencing using ABI BigDye (Applied Biosystems, Foster City, CA 94404).

## Authors' contributions

FJ, JC and LK conceived the project. JC, LW, LK performed the experimental research. JC and FJ wrote the manuscript with input from all the authors. All authors read and approved the final manuscript.
